# Adenomatous hyperplasia induced by chronic cherry pit retention mimicking an endobronchial tumor-case series and systematic review of literature

**DOI:** 10.3389/fmed.2024.1404951

**Published:** 2024-07-17

**Authors:** Gani Oruqaj, Gabriele Krombach, Stefan Gattenloehner, Susanne Herold, István Vadász, Werner Seeger, Khodr Tello, Matthias Hecker

**Affiliations:** ^1^Department of Internal Medicine, Medical Clinic II, Universities of Giessen and Marburg Lung Center (UGMLC), Institute for Lung Health (ILH), Cardio-Pulmonary Institute (CPI), Member of the German Center for Lung Research (DZL), Giessen, Germany; ^2^Department of Radiology, University Hospital Giessen, Giessen, Germany; ^3^Department of Pathology, Justus-Liebig- University Giessen, Universities of Giessen and Marburg Lung Center (UGMLC), Giessen, Germany; ^4^Department of Internal Medicine V, Universities of Giessen and Marburg Lung Center (UGMLC), Institute for Lung Health (ILH), Cardio-Pulmonary Institute (CPI), Member of the German Center for Lung Research (DZL), Giessen, Germany

**Keywords:** adenomatous hyperplasia, squamous metaplasia, actinomycosis, cherry pit, mimicking lung

## Abstract

**Introduction:**

Endobronchial foreign body aspiration is not common in adults, but it is a life-threatening event. Recurrent pneumonias by chronic retention of foreign body often lead to initial medical presentation of the patient. However, lymphoplasmacellular bronchitis with adenomatous hyperplasia and squamous epithelium metaplasia with complete or partial blockage of lobar bronchus mimicking lung tumor is rare in literature, and this particular condition is often misdiagnosed.

**Case presentation:**

we report our experience in the diagnostic and management of two elderly patients with recurrent pneumonia, admitted in hospital for further examination. In both patients, with no history of aspiration, the cherry pit was detected during bronchoscopy and recanalization with flexible cryoprobe, surrounded by purulent secretion, occluding completely the right upper lobe in the first case, and partially the left lower lobe associated with persistent actinomycosis in the second case, with signs of local inflammation, bronchial adenomatous hyperplasia mimicking lung tumor at initial bronchoscopic examination. Histology showed a lymphoplasmacellullar bronchitis with adenomatous hyperplasia and squamous epithelium metaplasia because of chronic retention of foreign body.

**Conclusion:**

Bronchoscopy examination should be considered in cases where there is an unresolved chronic cough with recurrent pneumonia or persistent actinomycosis in patients with high risk. Cryoprobe is a safe and feasible approach for treatment of airway obstructions due to chronic foreign body retention. Furthermore, relevant findings are discussed here, along with a review of the pathologic alterations and treatment modalities seen in chronic retention of foreign body and airway injury.

## Introduction

Recurrent Pneumonia due to chronic retention of foreign body, which mimics a bronchial tumor is a rare observed condition in adults (1). Bronchoscopy is an uncommon indication for foreign body aspiration in adults, accounting for 1% of procedures (2). The diagnosis of the foreign body aspiration is often delayed or overlooked, leading to patients experiencing chronic cough, recurrent pulmonary infections and persisting dyspnea (3, 4). Cryotherapy with flexible cryoprobe is a safe technique for treatment of symptomatic endobronchial tumor stenosis, cryoextraction and quick removal of foreign bodies (5–8). Furthermore, actinomycosis associated with foreign body aspiration is a rare pulmonary infection, caused by inhalation of actinomyces contaminated aspiration (9).

Here, we report two unique cases of cherry pit aspiration, who unconsciously aspirated a cherry pit months or years prior to recurrent pulmonary infections. The patients were not aware of any aspiration. Bronchoscopy is the recommended initial management and diagnostic procedure for patients experiencing recurrent pulmonary infections, unresolved actinomycosis resistant to antibiotics regardless whether or not they have a history of aspiration.

### Case 1 presentation

**Figure 1:**
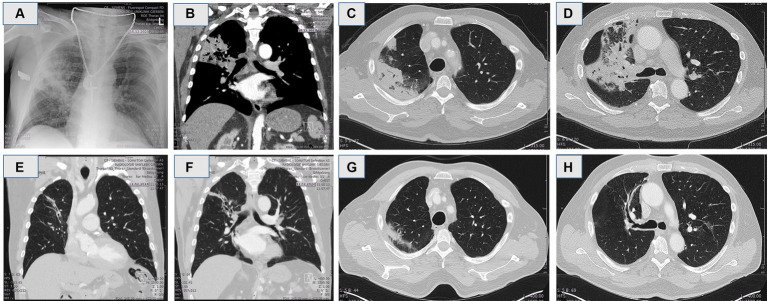
**(A)** Chest X-ray showing a consolidation in the right upper lobe indicating of pneumonia. **(B–D)** Computerized tomography (CT) in coronal and axial reconstruction demonstrated compression of the upper right bronchus and post-stenotic pneumonia of right upper lobe resembling lung cancer. **(E–H)** Computerized tomography in coronal and axial reconstruction 8 weeks post extraction of the cherry pit.

A 70-year old male patient with past medical history of hypertension, asthma, cardiovascular disease was admitted on December 14, 2023, because of persistent respiratory symptoms with intermittent coughing, dyspnea, fever in the last 2 days, and prior history of Pneumonia 2 weeks before admission which was treated with antibiotics. There was a slight improve after therapy, with persisting symptoms afterwards. Upon inquiry about aspiration, a suspicion of a foreign body in the bronchi was raised, in addition to suspicion of a tumor occlusion. History of choking due to foreign body aspiration and allergy were denied, but in last months has become more forgetful. Physical examination at admission showed a body temperature of 38°C, pulse rate of 89 beats per minute, blood pressure 113/78 mmHg, and respiratory rate of 29 breaths per minute, oxygen saturation on room air 92% with 2 L O_2_. The patient had a clear consciousness. Physical examination appeared unremarkable, lungs with no wheezing, but diminished breath sounds on right upper side. The chest-X ray and a low dose CT scan demonstrated consolidation of the right upper lobe, with compression of the upper bronchus and poststenotic pneumonia and suspicious diagnosis of lung cancer (Figures 1A–D). A sputum analysis revealed a normal respiratory microbiota. Biochemical analysis revealed elevated C reactive protein of 159 mg/L (reference value <5 mg/L) and a white cell count of 10.3×10 9/l (reference value 3,9–10 giga/l). Renal function test normal, while liver function test slightly increased liver enzymes (GOT, GPT). C reactive protein (CRP) after therapy recovered and was again normal.

**Figure 2:**
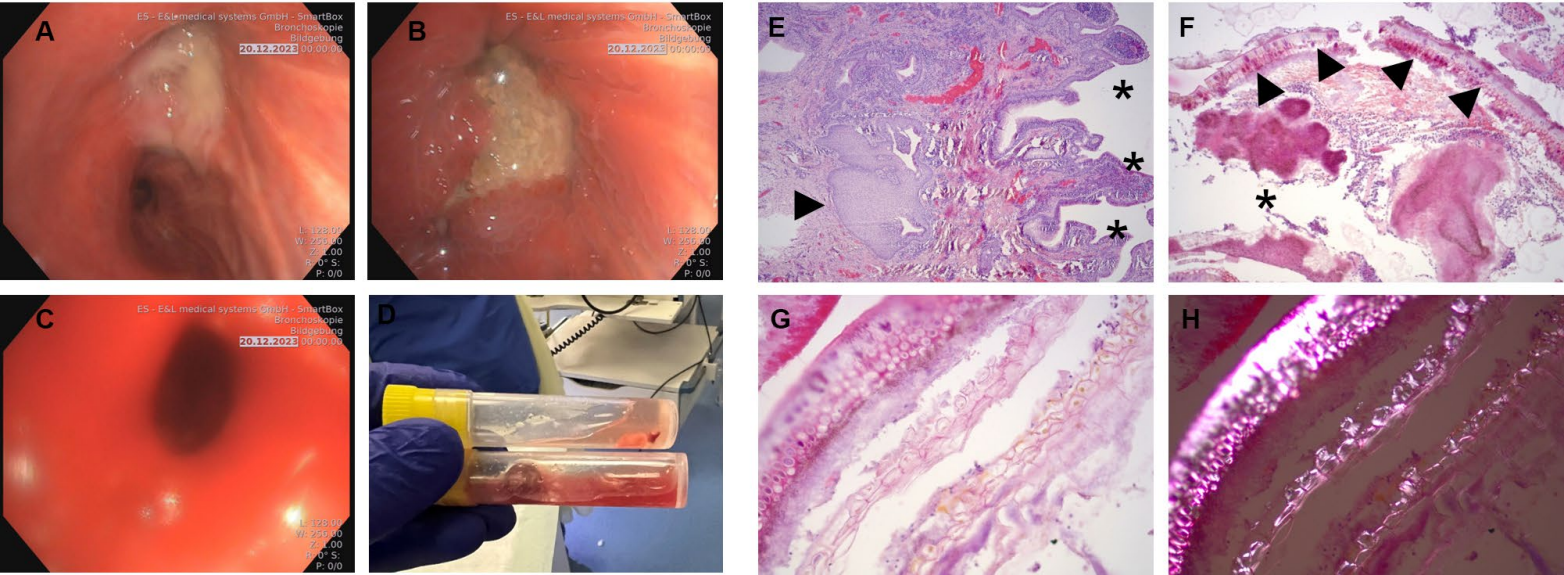
**(A,B)** Flexible bronchoscopy revealing total obstruction of the right upper lobar bronchus by amorphous bronchial adenomatous hyperplasia, purulent secretion and abundant granulation tissue. **(C)** The right upper lobar bronchus after retrieval of the foreign body and adenomatous hyperplasia by flexible cryoprobe. **(D)** Macroscopic appearance of the aspirated material cherry pit with hyperplasia tissue and purulent secretions in a specimen container, after removal with cryoprobe. **(E)** Pathological examination of bronchial epithelial hyperplasia with reactively inflamed bronchial mucosa with chronic bronchitis with pseudopapillary epithelial hyperplasia (stars) and pronounced squamous epithelium metaplasia (arrow) (**H**&**E**, 20x). **(F)** Immunostaining of plant material (arrows) and secondary bacterial colonization by coccoid and filamentous bacteria (star) (**H**&**E**, 50x). **(G,H)** Immunostaining of detailed typical water-clear plant cells **(G)**, polarized microscope image **(H)** with very characteristic birefringence as evidence of plant foreign material (**H**&**E**, 200x).

As next, the patient underwent fiberoptic bronchoscopic exploration with appropriate sedation using midazolam, propofol and hydromorphone, which lasted approximately 30 min. Flexible bronchoscopy showed a complete occlusion of the right upper lobar bronchus by adenomatous hyperplasia tissue and purulent secretion resembling macroscopically a lung tumor (Figures 2A–D). Purulent secretion distal to obstruction was cleared with suction, and hyperplasia tissue was carefully removed with cryoprobe, and finally cherry pit surrounded with epithelium hyperplasia and purulent secretion was successfully extracted with a cryoprobe (see Supplementary Video S1). Airways distal to obstruction were markedly bronchiectatic and altered (Figure 1). To our knowledge, this is the first case report regarding aspiration of cherry pit and subsequent pathologic-histologic alterations with adenomatous hyperplastic bronchitis, epithelium metaplasia mimicking a lung tumor.

Eight weeks after removal of the foreign body and adenomatous tissue, the patient underwent a follow-up CT scan that showed no longer consolidation of the right upper lobe, with minor bronchiectasia distally (Figures 1E–H).

The pathological examination of the tissue and foreign body revealed adenomatous hyperplasia and lympoplasmaceullar bronchitis with squamous epithelial metaplasia in the extracted tissue due to cherry pit aspiration, with no signs of dysplasia in the examined specimen (Figures 2E–H). The bronchial epithelial is reactively inflamed with chronic bronchitis and pseudopapillary epithelial hyperplasia and pronounced squamous epithelium metaplasia (Figure 2). The plant material (cherry pit) and secondary bacterial colonization by coccoid and filamentous bacteria could be well demonstrated (Figures 2A–H). Moreover, typical water-clear plant cells with very characteristic birefringence providing evidence of plant material, could be well observed with the use of polarized microscope (Figures 2G,H). Furthermore, in the final figure, the presence of a cherry pit foreign body within a specimen container, along with purulent secretions and bronchial hyperplasia tissue, was clearly visible (Figure 2D).

### Case 2 presentation

A 74-year old patient with a history of COPD GOLD III, pulmonary hypertension presented with a productive cough yielding yellow sputum that had worsened over the past 8 weeks. The patient also reported generalized body weakness and a decreased appetite with the acute illness, but no fever or chills. The patient has intermittently symptoms in the past 2 years. Initially, there was performed a bronchoscopy fur further evaluation and diagnostics, which revealed a tumor like mass in the 8th segment of the left lower lobe. An endobronchial biopsy of the lesion showed an epithelium metaplasia, and revealed filamentous gram-positive organisms resembling actinomyces infection, which was treated with penicillin, and made good clinical improvement with radiological resolution.

**Figure 3:**
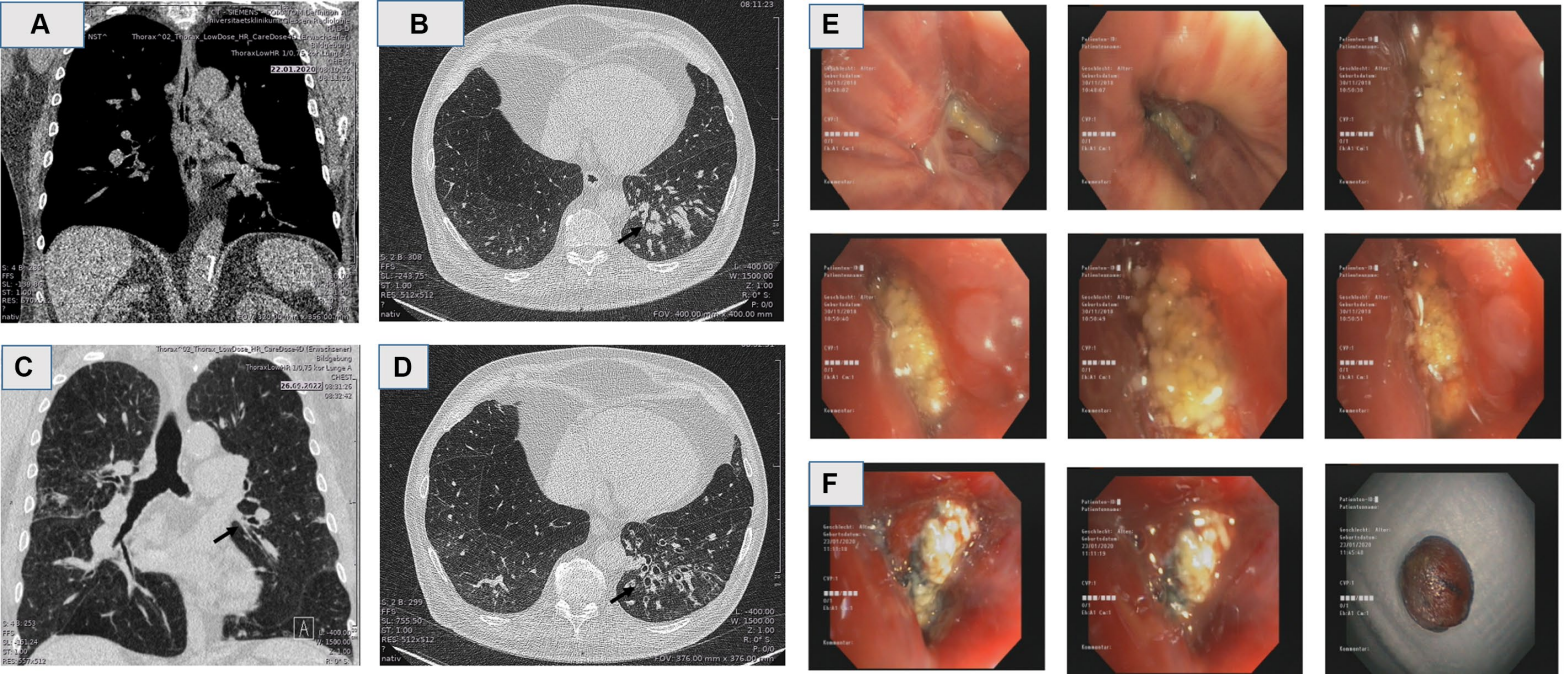
**(A,B)** Computerized tomography (CT) in coronal and axial reconstruction demonstrated compression of the lower left bronchus, followed by mucus obstruction of the bronchi, and post-stenotic bronchiectatic actinomycosis. **(C,D)** Computerized tomography in coronal and axial reconstruction in follow up, months post extraction of the cherry pit, revealing complete resolution with remaining bronchiectasia distally. **(E,F)** flexible bronchoscopy of the left lower lobe at initial presentation revealed an actinomycosis in biopsy **(E)**, and remaining obstruction in 2 years follow up, with the macroscopic appearance of the aspirated material, mimicking lung tumor **(F)**.

At presentation 2 years later, he gave 8-week history of increasing breathlessness on exertion and cough productive of purulent sputum. He denied any haemoptysis, chest pain or weight loss but complained of episodic sweating three times per week. He was afebrile, with a pulse of 76 beats per minute and oxygen saturations of 91 percent on room air. White blood cell count was normal at 4.4 × 109/L. Chest radiograph revealed left lower zone consolidation with evidence of collapse (Figure 3A). Subsequent contrast enhanced computerized tomography (Figures 3A,B) demonstrated partial occlusion of left lower lobe with mucous secretion, bronchiectatic and altered, indicating for an actinomycosis infection.

Again, the flexible bronchoscopy examination was performed by using sedation with midazolam, propofol and hydromorphone, which lasted approximately 30 min. It revealed further the adenomatous hyperplasia and partial occlusion of the segment 8 in the left lower lobe. A grey tumor-like mass in bronchus 8th segment was noted. The mass was exerted with flexible cryoprobe (Figures 3E,F). Histology revealed chronically inflamed mucosa, cherry pit foreign material and filamentous gram-positive organisms resembling actinomyces, after years of latency.

In the follow-up CT scan after removal of the foreign body and adenomatous tissue, showed no longer consolidation of the left lower lobe, with remaining bronchiectasia distally (Figures 3C,D).

## Discussion

Foreign body aspiration (FBA) is an uncommon event in the adult population. In these case series study, the patient in case 1 presented with symptoms of pneumonia, and his chest CT scan revealed a consolidation in the right upper lobe, and in case 2 presented with persistent actinomycosis infection resistant to antibiotics and dyspnea in exertion, in both cases resembling an endobronchial tumor. Since the recurrent pneumonia and persistent actinomycosis were the main reasons for admission to the hospital, we performed a bronchoscopy for further diagnostical purpose. However, in case 1 the patient can remember eating cherries 6–8 months before admission to hospital, but did not notice any aspiration of it, whereas in case 2 the patient did not notice any aspiration, after a long latency of the cherry pit. We suppose that the aspiration symptoms were mild due to their elderly and poor general condition. In case 1 during bronchoscopy showed a complete occlusion of the right upper lobar bronchus and hyperplasia tissue covered with purulent secretion resembling an endobronchial tumor. The obstruction including epithelium hyperplasia was carefully removed with a cryoprobe (Figure 2D; Supplementary Video S1). In case 2, in bronchoscopic examination revealed a partial occlusion of the left lower lobe with a tumor mass, hyperplasia tissue covered with purulent secretion mimicking also a lung tumor (Figures 3A–F), which was carefully removed with a cryoprobe (Figure 3F). Foreign body aspiration usually occurs in children, and the other peak at over 60 years of age accounting for 20% aspiration cases (10, 11). A foreign body aspiration can be tolerated in the elderly ages leading to recurrent pneumonia, and patients presenting with symptoms due to infection and other complications such as pneumothorax, or endobronchial tumor (12). Colleagues Li et al. prescribed a case report of watermelon seed aspiration in a cancer patient resembling a recurrence (3). Furthermore, actinomycosis is a purulent infection caused by filamentous bacilli, and pulmonary actinomycosis presented only 15% of actinomyces infection (13). Pulmonary actinomycosis is presented in various form, including pneumonia, a pseudo-tumor-like mass, draining fistulas, pulmonary abscess or tuberculosis (14, 15). Actinomycosis clinical cases due to nonorganic FBA have been reported in the literature such as secondary to pen end cap, or watermelon seed aspiration (16, 17).

However, there are no reports of cherry pit aspiration and adenomatous hyperplasia with lymphoplasmacellular bronchitis, squamous epithelium metaplasia, actinomycosis infection mimicking also macroscopically a lung cancer.

Foreign body aspirations are often characterized with histopathologic sequela of chronic inflammatory changes, including lymphocyte and plasma cell infiltration, fibrosis, and the formation of foreign body giant cells and granulomas (18). The changes resembling a tumor mass were seen in our clinical case, adenomatous hyperplasia adjacent to the aspirated cherry pit, which also revealed in addition squamous metaplasia. Secondary bacterial colonization by coccoid and filamentous bacteria were observed in H&E staining (Figure 2F). Interestingly in pathological specimen was observed clearly typical water-clear plant cells, with typical birefringence as evidence of foreign plant material (Figures 2E–H). Furthermore, a macroscopic view of a cherry pit foreign body could be finally observed in a specimen container (Figures 2D, 3F). Squamous epithelium metaplasia is normally seen as secondary to the toxic injury of the airways as precursor of squamous cell carcinoma (19). Squamous metaplasia has been documented to happen in smokers with chronic obstructive pulmonary disease, due to chronic toxic injury, expressing carcinoembryonic antigen (19). Additionally, epithelium squamous metaplasia possibly caused by aspiration of a pistachio shell, was detected during a CT-Screening for lung cancer, and supported in PET-CT scan for lung tumor (20). Similar cases of chronic inflammation and induction of squamous metaplasia due to retention of oral chaff debris in rat feed were observed in animal models (21). This phenomenon is also presented in our cases, in which adenomatous hyperplasia and epithelium squamous metaplasia were observed in addition to the chronic inflammation, purulent secretion and aspirated cherry pit. Both chronic smoking and FBA have been shown to induce epithelium squamous metaplasia, it might be unclear if the cherry pit is only responsible factor, and smoking is additional factor contributed to the event. Juan et al. showed that DNA damage has the potential to drive the squamous metaplasia via mitotic checkpoints, opening novel molecular candidate targets for lung squamous cell carcinoma (22). Furthermore, atypical adenomatous hyperplasia is prescribed as risk factor for lung cancer (23).

Once the FBA is suspected with detailed clinical history and imaging findings, bronchoscopy is the next step in management of foreign body retrieval. Flexible bronchoscopy is a valuable procedure, with less discomfort, performing under light sedation, and enables access to the peripheral airways in medical centers where rigid bronchoscopy is not available (24). However, rigid bronchoscopy has been favored historically for foreign body removal in the pediatric population.

Cryotherapy with flexible cryoprobe is a safe technique for treatment of symptomatic endobronchial tumor stenosis, cryoextraction and quick removal of foreign bodies (5–8).

Cryobiopsy has emerged as a safe and effective method in diagnostics, with additional benefits over conventional biopsy when used in combination with radial endobronchial ultrasound (25, 26). Furthermore, cryoprobe plays a crucial role in the cryorecanalization of malignant airway obstruction, leading to improved patient survival (27). Additionally, use of cryoprobe has been proven safe and feasible for the retrieval of both organic and inorganic foreign body aspirations, providing a versatile tool for medical professionals (7).

The pneumonia in the follow-up CT in both cases were completely resolved (Figures 1E–H, 3C,D). Minor changes with persistent bronchiectasis in follow-up CT, are most likely in resolving process of pneumonia, or in chronic latency in case 2 associated with COPD.

In case 2 the tumor mass associated with pulmonary actinomycosis reported here was most likely due to the aspiration of the cherry pit, which had been present in the lung for a long duration, *ca.* 2 years. This indicates in addition, the importance of keeping actinomycosis of the lung in the differential diagnosis of pseudo-tumor of a lung lesion, a bronchoscopy after resolution of the local inflammation is imperative, and samples should be also for tissue diagnosis and regular culture. In elderly patients with aspiration risk, prevention of aspiration with upright positioning during meals, supervision from nurses, and routine oral care are crucial factors (28).

In conclusion, bronchoscopy examination should be considered as a diagnostic tool in cases of recurrent pneumonia or persistent actinomycosis that are resistant to antibiotics, to exclude any chronic foreign body retention as a potential underlying cause of the event. In addition, cryoprobe is safe, efficient and feasible method in retrieval of bronchial adenomatous hyperplasia resulting from chronic foreign body retention. This clinical novel rarity, with recurrent pneumonia or endobronchial actinomycosis associated with foreign body aspiration, which mimics a lung tumor with well-defined edges, requires special attention from medical staff in specific situations.

## Data availability statement

The original contributions presented in the study are included in the article/Supplementary material, further inquiries can be directed to the corresponding author/s.

## Ethics statement

The studies involving humans were approved by Justus Liebig University Medizinisches Lehrzentrum. The studies were conducted in accordance with the local legislation and institutional requirements. The participants provided their written informed consent to participate in this study. Written informed consent was obtained from the individual(s) for the publication of any potentially identifiable images or data included in this article.

## Author contributions

GO: Conceptualization, Data curation, Formal analysis, Investigation, Writing – original draft, Writing – review & editing, Methodology, Software, Validation, Visualization. GK: Data curation, Formal analysis, Investigation, Writing – review & editing. SG: Formal analysis, Investigation, Resources, Writing – review & editing. SH: Data curation, Formal analysis, Investigation, Writing – review & editing. IV: Data curation, Formal analysis, Investigation, Writing – review & editing. WS: Conceptualization, Investigation, Supervision, Writing – review & editing. KT: Data curation, Formal analysis, Investigation, Supervision, Validation, Writing – review & editing, Writing – original draft, Conceptualization, Visualization. MH: Data curation, Formal analysis, Investigation, Supervision, Validation, Writing – review & editing, Writing – original draft, Conceptualization, Methodology, Visualization.

## Supplementary material

SUPPLEMENTARY VIDEO S1 Video of the flexible bronchoscopy: retrieval of the adenomatous hyperplasia and cherry pit foreign body.

## References

[1] Bajaj D Sachdeva A Deepak D. Foreign body aspiration. J Thorac Dis. (2021) 13:5159–75. doi: 10.21037/jtd.2020.03.94, PMID: 34527356 PMC8411180

[2] Hewlett JC Rickman OB Lentz RJ Prakash UB Maldonado F. Foreign body aspiration in adult airways: therapeutic approach. J Thorac Dis. (2017) 9:3398–409. doi: 10.21037/jtd.2017.06.137, PMID: 29221325 PMC5708401

[3] Li L Li MJ Sun L Jiang YL Zhu J. Neglected foreign body aspiration mimicking lung Cancer recurrence. Risk Manag Healthc Policy. (2022) 15:491–6. doi: 10.2147/RMHP.S361081, PMID: 35321269 PMC8935719

[4] Madsen A Madsen PH. Recurrent pneumonia due to endobronchial foreign body. BMJ Case Rep. (2014) 2014:bcr2013201959. doi: 10.1136/bcr-2013-201959, PMID: 24994763 PMC4091130

[5] Hetzel M Hetzel J Schumann C Marx N Babiak A. Cryorecanalization: a new approach for the immediate management of acute airway obstruction. J Thorac Cardiovasc Surg. (2004) 127:1427–31. doi: 10.1016/j.jtcvs.2003.12.032, PMID: 15116003

[6] Schumann C Hetzel M Babiak AJ Hetzel J Merk T Wibmer T. Endobronchial tumor debulking with a flexible cryoprobe for immediate treatment of malignant stenosis. J Thorac Cardiovasc Surg. (2010) 139:997–1000. doi: 10.1016/j.jtcvs.2009.06.023, PMID: 19716140

[7] Fruchter O Kramer MR. Retrieval of various aspirated foreign bodies by flexible cryoprobe: in vitro feasibility study. Clin Respir J. (2015) 9:176–9. doi: 10.1111/crj.12120 24521482

[8] Sriratanaviriyakul N Lam F Morrissey BM Stollenwerk N Schivo M Yoneda KY. Safety and clinical utility of flexible Bronchoscopic Cryoextraction in patients with non-neoplasm tracheobronchial obstruction: a retrospective chart review. J Bronchol Interv Pulmonol. (2015) 22:288–93. doi: 10.1097/LBR.0000000000000203, PMID: 26439016

[9] Chouabe S Perdu D Deslée G Milosevic D Marque E Lebargy F. Endobronchial actinomycosis associated with foreign body: four cases and a review of the literature. Chest. (2002) 121:2069–72. doi: 10.1378/chest.121.6.2069, PMID: 12065381

[10] Tseng HJ Hanna TN Shuaib W Aized M Khosa F Linnau KF. Imaging foreign bodies: ingested, aspirated, and inserted. Ann Emerg Med. (2015) 66:570–582.e5. doi: 10.1016/j.annemergmed.2015.07.499, PMID: 26320521

[11] Baharloo F Veyckemans F Francis C Biettlot MP Rodenstein DO. Tracheobronchial foreign bodies: presentation and management in children and adults. Chest. (1999) 115:1357–62. doi: 10.1378/chest.115.5.1357 10334153

[12] Ha JH Jeong BH. Airway foreign body mimicking an endobronchial tumor presenting with pneumothorax in an adult: A case report. Medicina (Kaunas). (2021) 57:50. doi: 10.3390/medicina57010050 33430107 PMC7827418

[13] Kononen E Wade WG. Actinomyces and related organisms in human infections. Clin Microbiol Rev. (2015) 28:419–42. doi: 10.1128/CMR.00100-14, PMID: 25788515 PMC4402957

[14] Kim SR Jung LY Oh IJ Kim YC Shin KC Lee MK. Pulmonary actinomycosis during the first decade of 21st century: cases of 94 patients. BMC Infect Dis. (2013) 13:216. doi: 10.1186/1471-2334-13-216, PMID: 23672372 PMC3658925

[15] Katsenos S Galinos I Styliara P Galanopoulou N Psathakis K. Primary bronchopulmonary Actinomycosis masquerading as lung Cancer: apropos of two cases and literature review. Case Rep Infect Dis. (2015) 2015:609637. doi: 10.1155/2015/609637 26146575 PMC4471307

[16] Singh S Singh S Jyotimallika J. Endobronchial actinomycosis associated with nonorganic foreign body aspiration after years of latency. J Bronchol Interv Pulmonol. (2015) 22:180–2. doi: 10.1097/LBR.0000000000000143, PMID: 25887022

[17] Kassab K Karnib M Bou-Khalil PK Bizri AR. Pulmonary actinomycosis presenting as post-obstructive pneumonia. Int J Infect Dis. (2016) 48:29–31. doi: 10.1016/j.ijid.2016.04.009, PMID: 27085876

[18] Bozkurtlar E Oksuzoglu K Bostanci K Aslan S Kissa TN Kocakaya D. FDG PET/CT features of polysaccharide-based hemostatic agent: chronic inflammatory changes can mimic metastatic lesions. Clin Nucl Med. (2022) 47:e475–80. doi: 10.1097/RLU.0000000000004216 35452003

[19] Rigden HM Alias A Havelock T O'Donnell R Djukanovic R Davies DE. Squamous metaplasia is increased in the bronchial epithelium of smokers with chronic obstructive pulmonary disease. PLoS One. (2016) 11:e0156009. doi: 10.1371/journal.pone.0156009, PMID: 27228128 PMC4881906

[20] Purohit K Grandfield S Dhamija A Abbasi A. Foreign body aspiration mimicking an endobronchial neoplasm: a case report and review of the literature. Cureus. (2023) 15:e36105. doi: 10.7759/cureus.36105, PMID: 37065369 PMC10098029

[21] Madsen C. Squamous-cell carcinoma and oral, pharyngeal and nasal lesions caused by foreign bodies in feed. Cases from a long-term study in rats. Lab Anim. (1989) 23:241–7. doi: 10.1258/002367789780810572, PMID: 2668638

[22] Juan LS Freije A Sanz-Gómez N Jiménez-Matías B Pleguezuelos-Manzano C Sanz JR. DNA damage triggers squamous metaplasia in human lung and mammary cells via mitotic checkpoints. Cell Death Discov. (2023) 9:21. doi: 10.1038/s41420-023-01330-3, PMID: 36681661 PMC9867756

[23] Wang GF Lai MD Yang RR Chen PH Su YY Lv BJ. Histological types and significance of bronchial epithelial dysplasia. Mod Pathol. (2006) 19:429–37. doi: 10.1038/modpathol.3800553, PMID: 16415791

[24] Ramos-Rossy J Cantres O Torres A Casal J Otero Y Arzon-Nieves G. Flexible Bronchoscopic removal of 3 foreign objects. Fed Pract. (2018) 35:24–6. PMID: 30766383 PMC6366794

[25] Thiboutot J Illei PB Maldonado F Kapp CM DeMaio A Lee HJ. Safety and feasibility of a sheath Cryoprobe for Bronchoscopic Transbronchial biopsy: the FROSTBITE trial. Respiration. (2022) 101:1131–8. doi: 10.1159/000526876, PMID: 36265451

[26] Nakai T Watanabe T Kaimi Y Shiomi K Ando K Miyamoto A. Diagnostic utility and safety of non-intubated Cryobiopsy technique using a novel ultrathin Cryoprobe in addition to conventional biopsy techniques for peripheral pulmonary lesions. Respiration. (2023) 102:503–14. doi: 10.1159/000531010, PMID: 37379810

[27] Ma Q Shi B Tian Y Liu D. Fibrobronchoscopic cryosurgery for secondary malignant tumors of the trachea and main bronchi. Thorac Cancer. (2016) 7:459–66. doi: 10.1111/1759-7714.12337, PMID: 27385989 PMC4930966

[28] Thomas LE Lustiber L Webb C Stephens C Lago AL Berrios S. Aspiration prevention: a matter of life and breath. Nursing. (2019) 49:64–6. doi: 10.1097/01.NURSE.0000552698.50502.75 30801411

